# A Case of Off-label Sodium Oxybate in an 11-Year-Old with Narcolepsy

**DOI:** 10.7759/cureus.4057

**Published:** 2019-02-12

**Authors:** Jeffrey A Miskoff, Moiuz Chaudhri

**Affiliations:** 1 Internal Medicine, Jersey Shore University Medical Center, Neptune City, USA

**Keywords:** case report, narcolepsy, pediatric, off label, sodium oxybate, hypocretin, sleep disorder, excessive sleepiness

## Abstract

Narcolepsy is a rare chronic condition which affects sleep architecture. It may manifest in childhood or adolescence by causing excessive daytime sleepiness, sleep attacks, and hallucinations. Research suggests that there is a significant delay in diagnosis with the mean age being 10 to 15 years from onset of symptoms. Although narcolepsy is predominantly associated with loss of hypocretin (orexin), the role of genetics is complementary to the diagnosis. In addition, clinical manifestations of symptoms vary in presentation and severity from case to case. Therefore, this report provides an opportunity to review pediatric narcolepsy including the diagnostic workup and clinical response to sodium oxybate. This particular case describes an 11-year-old boy meeting the clinical and diagnostic criteria for narcolepsy. Clinically, the patient had a very positive response to treatment with sodium oxybate, which at the time of initiating therapy was off-label for patients under the age of 18.

## Introduction

Narcolepsy is a sleep disorder which impairs sleep-wake cycles. Although manifestation of symptoms may vary from patient to patient, its impact on the patients and their families may be significant especially before an official diagnosis is made. Excessive daytime sleepiness (EDS) is the most prevalent symptom which can occur in isolation or combination with features typically seen in disorders of rapid eye movement (REM), cataplexy, hallucinations, and sleep paralysis [1]. Evidence suggests that a deficit of hypocretin (neuropeptide) is the main culprit behind the genesis of this condition [2]. The primary focus of this case study is to 1) highlight the importance of continued education to improve the time to a correct diagnosis and 2) review current therapies and their efficacy.

## Case presentation

An 11-year-old African-American male presented to our care in January 2011 because of excessive daytime sleepiness and episodes of losing muscle tone upon experiencing strong emotional stimuli. Before seen by us, the patient was evaluated by a psychiatrist in March 2010 at a request of his mother. Clinician records suggest that the patient was being seen for not being able to express himself, labile mood, lack of focus and concentration at school, anxiety, and lack of self-esteem. At the end of the evaluation by the psychiatrist, the patient was diagnosed with Adjustment disorder of childhood with mixed emotions, depressed feelings, and attention deficit hyperactivity disorder primarily inattentive type. Furthermore, the patient scored 55 (normal = 91-100) on the global assessment of functioning (GAF) score. It is a numerical scale utilized by mental health clinicians to determine the patient's day to day functionality. Specifically, it allows the clinicians to score and determine their social, occupational and psychological functioning [3].

After presenting to our care, the patient was sent for further workup including the Epworth Sleepiness Scale (ESS), a nocturnal polysomnogram (NPSG), and a multiple sleep latency test (MSLT). The ESS is a subjective test utilized to measure the patient’s sleepiness [4]. It includes eight scenarios in which the patient rates their tendency to become sleepy. The scale ranges from 0 (no chance of dozing) to 3 (high chance of dozing). These eight scenarios are sitting and reading, watching television, sitting inactive in a public place, as a passenger in a car for an hour without a break, in a car while stopped for a few minutes in traffic, lying down to rest in the afternoon, sitting and talking to someone, and sitting quietly after a lunch without alcohol [4, 5]. Our patient scored 17 which is highly associated with pathologic sleepiness because a score of greater than 15 suggests that the patient is excessively sleepy and should be treated.

NPSG is a sleep study which records brain waves, oxygen levels, heart rate and breathing patterns along with eye and extremity movement as the patient sleeps [6]. According to the results of NPSG, the patient slept 367.00 minutes out of 440.7 minutes in bed for a sleep efficiency of 83.3%. The patient spent 66.4% of total sleep time in supine position. In addition, the patient spent 16.9%, 38.3%, 27.5% of sleep time in stage 1, 2 and 3, respectively. The study identified five REM sleep periods with REM stage lasting for 17.3% of sleep time. REM latency was recorded as normal at 87.5 minutes. Furthermore, the sleep study noted sleep latency (SL) to be 3.2 minutes. Overall Apnea-Hypopnea Index was 0.5 events/hour. The REM specific index was 1.9 events per hour. During the study, one obstructive hypopnea was with a mean duration of 14.3 seconds. There were two mixed apneas with a mean duration of 10.7 seconds along with 0 central apneas. Laboratory workup for HLA-DR15 and DQ0602 was positive.

MSLT is the primary diagnostic tool for narcolepsy and is typically performed following an NPSG to measure sleep latency [7]. Sleep latency can be described as the amount of time it takes to go from wakefulness to entering sleep. Also, it measures sleep onset REM periods (SOREMPs) which illustrates how quickly the patient enters REM sleep [7]. This test typically includes four or five naps lasting for 20 minutes each. Results of the MSLT consisted of five nap trials with a sleep latency of 1.8, 3.4, 4.7, 2.9, 6.4 minutes, respectively. In addition, REM latency during five nap trials was recorded to be at 2.5, 1.0, 1.5, 0.5 and 2.5 minutes, respectively. The result shows that the patient slept during each nap trial with mean sleep latency (MSL) to be at 3.8 minutes. Lastly, the study recorded five SOREMPs with a mean REM latency of 1.6 minutes. Based on the workup, the patient was diagnosed with narcolepsy which has been successfully managed with stimulant therapy and sodium oxybate.

## Discussion

Narcolepsy is one of the most commonly missed causes of excessive daytime sleepiness presenting with multifactorial etiology. Although the loss of hypocretin is the main culprit behind this condition, the role of genetics and infections is less well understood and therefore is complementary to the diagnosis [8]. Hypocretins, neurotransmitters transmitting chemical signal, are produced in neurons found in the hypothalamus, located roughly behind the eyes and between the ears. These neurotransmitters act primarily as excitatory stimuli, are released during wakefulness and bind to specific receptors leading to maintaining alertness and preventing REM sleep from occurring at the wrong time [9].

We presented a case of an 11-year-old male who presented to our care for the evaluation of excessive daytime sleepiness along with occasional cataplexy symptoms. The diagnostic criterion requires MSL of ≤8 minutes and ≥2 SOREMPs on MSLT [10]. Since our patient satisfied MSL (3.8 minutes) and SOREMP (5) components on three naps, continuing the study was not needed. Evidence suggests a strong association between narcolepsy and human leukocyte antigen (HLA) especially HLA-DQB1*0602 [11, 12]. In our patient, laboratory workup for HLA-DR15 and DQ0602 was positive, thus further supporting the diagnosis of narcolepsy. The family was relieved upon learning about the condition and its management which involves a combination of medications and behavior therapy. Therefore, the patient and the family were counseled on the benefits of regular behavioral therapy. It was explained that the behavioral education could help them learn about some of the activities that should be avoided. Specifically, activities such as riding a bicycle, swimming and any activity which can cause to lose muscle tone should be avoided.

In early November 2011, the patient was started on methylphenidate hydrochloride 10 milligrams (mg) extended release to be taken in the morning along with methylphenidate 5 mg to be taken in the late afternoon. Office records from December 2011 suggest that the symptoms of hypersomnia were not controlled with methylphenidate hydrochloride. In addition, these medications are not effective against cataplexy. In light of lacking therapeutic properties for our patient, options of adding venlafaxine either in isolation or in combination with sodium oxybate at a very low dose were discussed with the mother. It was explained that sodium oxybate and venlafaxine use would be off-label because these are not indicated to be used in patients younger than 18 years of age at the time of presentation, received Food and Drug Administration (FDA) approval in October 2018. At that time the patient was started on the lowest dose of sodium oxybate with the intention of titrating up based on his weight to reach a therapeutic dose. The patient was seen by the physician on a regular basis; records suggest that the patient reached a therapeutic dose of 2.5 grams taken twice for a total dose of 5 grams. During a recent visit, the patient expressed a desire to get his driving license. Therefore, he was sent for maintenance of wakefulness test (MWT) to quantify his ability to remain awake during a four to five sessions of 40 minutes in a quiet stimulation-free environment [13]. The patient must stay awake for 40 minutes during each trial which was not achieved by our patient. The patient indicated that he forgot to take his medication which might have contributed to not being able to stay awake during the MWT. The patient was counseled on sleep hygiene and the importance of taking his medications regularly. The patient will be retaking the MWT in 2019.

There are many reasons for presenting this case, the most important is to bring awareness about this condition. Literature suggests that most cases of pediatric patients are misdiagnosed or underdiagnosed despite presenting with the most common symptom of narcolepsy, excessive daytime sleepiness [8]. Furthermore, the data suggests that on average a patient visits numerous physicians before receiving a diagnosis of narcolepsy [14, 15]. A recent study suggests that annual direct medical costs associated with narcolepsy and matched cohort of patients without narcolepsy are $11,702 and $5,261 (p < 0.0001), respectively [15].

Although existing medications are effective in managing narcolepsy, ongoing research presents with Pitolisant, an inverse agonist of the histamine 3 (H3) receptor [16]. It was approved in March 2016 to treat narcolepsy in the European population, and Phase III Harmony trial shows that Pitolisant reduced daytime sleepiness along with improving attention [16]. In a different study, data suggest that opiates are capable of increasing the number of hypocretin-producing cells in humans and mouse brain along with reversing cataplexy in a mouse model [17].

## Conclusions

Narcolepsy is a chronic debilitating condition which is often undiagnosed or misdiagnosed due to its rarity. Although the most common symptom is excessive daytime sleepiness, other symptoms such as hallucinations, sleep paralysis with vivid dreams and cataplexy also manifest. A patient presenting with unexplained excessive daytime sleepiness should be investigated for this condition. Timely diagnosis and management can significantly improve patients’ quality of life and provide them with the opportunity to succeed. Since meeting this patient, sodium oxybate has received approval from the FDA for narcolepsy in pediatric patients aged 7-17 years.
